# Engineering a Coiled-Coil Protein for DARPin Presentation as a Potent SARS-CoV‑2 Therapeutic

**DOI:** 10.1021/acs.biomac.5c00593

**Published:** 2025-09-08

**Authors:** Linh D. Mai, Narayanaiah Cheedarla, Siamalan Krishnathas, Ishmamul H. Sadab, Mikaela A. Gray, Wei Lv, Anshul Dhankher, John D. Roback, Andrew S. Neish, Julie A. Champion

**Affiliations:** † School of Chemical and Biomolecular Engineering, 1372 Georgia Institute of Technology, 950 Atlantic Dr NW, Atlanta, Georgia 30332, United States; ‡ School of Materials Science and Engineering, Georgia Institute of Technology, 771 Ferst Drive, Atlanta, Georgia 30332, United States; § School of Medicine, 12239 Emory University, 100 Woodruff Circle, Atlanta, Georgia 30322, United States

## Abstract

The COVID-19 pandemic has demonstrated the need for rapid, flexible, and readily adaptable treatment options for future pandemic preparedness. Due to the speed at which viruses like SARS-CoV-2 mutate, the customary approach of using highly specific monoclonal antibodies as neutralization therapies is challenging, given their size, production complexity, and cost. Here, we leveraged rational protein design to create fusion proteins from small, antibody-mimetic proteins, Designed Ankyrin Repeat Proteins (DARPins) and a self-assembling hexameric coiled coil (CC-HEX). The fusion proteins are modular, suitable to rapidly adapt to new variants or pathogens, and enable the incorporation of both viral and serum albumin-binding functions. We demonstrated potent neutralization by HEX-DARPins against multiple variants of the SARS-CoV-2 pseudovirus. Albumin binding prolonged serum concentration and improved delivery to the lungs in mice. This work establishes HEX-DARPin fusion proteins as potential therapeutics for the treatment of COVID-19 and as a platform for the development of drugs against future viral pathogens.

## Graphical abstract

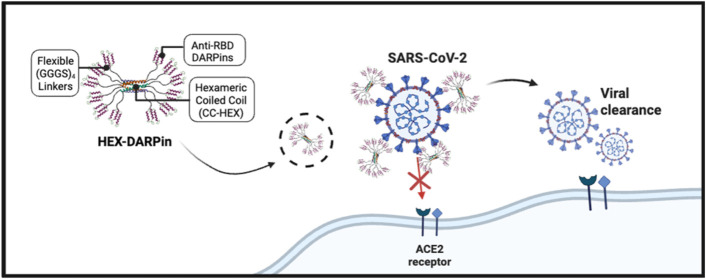

## 1 Introduction

According to the World Health Organization (WHO), as of November 2024, more than 7 million deaths have resulted from COVID-19 and its complications.^1^ Unfortunately, with climate change and a rapid rate of urbanization, another zoonotic, pandemic-level virus is inevitable. In fact, the chance of devastating pandemics, such as SARS-CoV-2 or the Spanish flu in 1918, is likely to grow 3-fold statistically in the coming decades.^2^ Therefore, the development of COVID-19 treatments is not only crucial for better outcomes for patients in the current pandemic but can also provide insight into guiding treatment development and resource allocation in preparation for future pandemics.

During infection, the virus first makes contact with the respiratory tract and infects the epithelial cells in the airway and lungs. Cellular entry of these viruses is facilitated through the interaction between the receptor binding domain (RBD) on the S1 subunit of its spike protein and the epithelial cell angiotensin converting enzyme 2 (ACE2).^3,4^ Inhibition of this RBD-mediated entry renders viruses unable to replicate.^3^ With the goal of keeping ACE2 and RBD apart, many molecules and macromolecules that are ACE2-targeting, ACE2-mimetic, or RBD-targeting were tested for COVID indication with varying levels of success.^5–9^ However, ACE2 is an important regulator of the vascular function with roles in the renin–angiotensin–aldosterone signaling pathway.^10^ Thus, blocking ACE2 or introducing recombinant ACE2 might be problematic, as it might affect vascular homeostasis and could lead to health complications, like hypertension.^11^

Alternatively, mimicking the natural immunological response, many recombinant monoclonal antibodies (mAbs) have been developed to bind to and block the viral RBD. Several of these have been approved for emergency use against COVID-19, such as Regeneron’s REGEN-COV, a mixture of casirivimab and imdevimab, or more recently, Bebtelovimab.^12–14^ Although being the gold standard for neutralizing molecules for their specificity and affinity to targets, antibodies are relatively large proteins (∼150 kDa) with disulfide-bond-rich structures and complicated post-translational modifications that require them to be expressed in mammalian cells.^15^ Despite many advances in the mammalian antibody expression workflow, it still lacks speed, cost-effectiveness, ease of use, and production capacity compared to bacterial expression systems.^16^ Additionally, the financial barrier resulting from proprietary materials, such as culture media, and greater labor requirements makes mammalian antibody expression less accessible to academic research,^16^ especially for those with more limited resources. This is nonideal, given that in a pandemic like COVID-19, speed and collaboration from scientific communities worldwide are critical for reducing fatalities and containing the disease.^17^ This problem calls for smaller and simpler binding proteins that can be rapidly produced in bacteria, making them more adept at keeping up with the new virus variants.

To address this need, multiple low-molecular-weight binders have been developed and studied for COVID-19 treatment, including nanobodies and affibodies.^18–21^ Among them, the binder that has reached clinical trials is the Designed Ankyrin Repeat Protein (DARPin), which has been engineered to create a class of antibody-mimetic proteins with high affinity, stability, and small size that allows easy modification and expression in *Escherichia coli*.^22,23^ Unlike some other popular classes of protein binders such as nanobodies, DARPins lack disulfide bonds, which allows them to be efficiently expressed in bacteria,^24^ without any further modification, additional expression complexity or specialized cell lines.^25–27^ The simple structure of DARPins, consisting of repeating motifs of β turns and antiparallel α helices, has enabled rapid screening and discovery, while also lowering their propensity for aggregation and, thus, immunogenic risk.^28,29^ Because of this, they have been implemented in multiple therapies, including immune-oncology,^30^ gene delivery^31^ and infectious diseases,^32^ showing their ability to be selected to bind to a wide range of targets. Particularly, many potent variants of DARPins against SARS-CoV-2 RBD have been identified^33^ and used in the design of clinical stage therapeutics, such as in the case of ensovibep by Molecular Partners.^34^ However, the use of monovalent DARPins often does not yield sufficient and sustained neutralization, which motivated the linear trivalent configuration of ensovibep or trimeric DARPins-foldon by Chonira et al.,^33^ though there has been proof that further increasing valency, to a hexameric configuration, in the low-valency regime will improve the avidity effect.^34–36^ Options to increase valency include higher multivalent delivery systems that could display many copies of affinity proteins for engagement, such as virus-like nanoparticles (VLPs). However, VLPs are known to be immunogenic,^37^ which is beneficial in vaccine applications, but might be detrimental in COVID-19 treatment, where it could exacerbate the already cytokine-rich environment. Furthermore, the high curvature of VLPs makes it unlikely that a significant number of binding proteins will engage with the same virus. The limitations of both linear and VLP multivalent designs motivated the exploration of a protein platform with properties between these two extremes.

CC-HEX is a *de novo* self-assembling parallel homohexameric coiled coil (PDB ID: 3R3K).^38^ With a molecular weight of 10.76 kDa, multivalency, and modular nature, CC-HEX possesses the potential to be an effective drug delivery and display platform for any single-chain therapeutic protein or peptide. Previous research in our group has successfully delivered several reporter and functional proteins intracellularly, including antibodies.^39–42^ However, we found that by distributing cargos on both termini of CC-HEX, cellular uptake could be avoided, keeping the carrier–drug complex in the extracellular space.^39^ The valency provided by the two termini of six α helices could greatly increase the avidity to targets, which is desirable for virus neutralization.^43^ With this in mind, we designed fusion proteins, HEX-DARPins, using CC-HEX as a carrier to display anti-RBD DARPins as the model small binding protein via long and flexible linkers, allowing a high level of cooperative available valency. We hypothesized that this fusion protein design, with increased avidity, would improve neutralization potency and protection across multiple variants of the SARS-CoV-2 virus. Additionally, we modified HEX-DARPins for a longer serum half-life by incorporating an albumin-binding DARPin^44^ on CC-HEX and studied the biodistribution of these molecules in a mouse model. This study marks the first time that CC-HEX, the first classical hexameric coiled-coil protein,^38^ has been successfully utilized as a delivery carrier *in vivo* and applied in the field of virus neutralization.

## 2 Materials and Methods

### 2.1 Protein Expression and Purification

Designed vector constructs for all fusion proteins were created by GenScript Inc. in pET-28A+ plasmids (see Table S1 for protein sequences). All vectors were transformed into heat-competent *E. coli* BL21 Star (DE3). Bacteria were cultured overnight in a 10 mL preculture of Luria–Bertani (LB) broth (ThermoFisher #12795084) containing kanamycin (0.05 g/L) at 37 °C, then added to 1 L of LB broth containing kanamycin and cultured at 37 °C until an OD_600_ of 0.6 was reached. Protein expression was induced by adding 1 mM isopropyl-β-d-1-thiogalactopyranoside for 3 h at 37 °C. The culture was then pelleted and resuspended in cold lysis buffer containing 10 mM imidazole, 50 mM NaHPO_4_, and 0.3 M NaCl, adjusted to pH 8. We purified the proteins under native conditions using Ni-NTA affinity chromatography according to the manufacturer’s protocol (Qiagen #30210). Gradient washing was done with a range of imidazole concentrations (20–60 mM) in wash buffers. Proteins were eluted from the Ni-NTA column with a buffer containing 250 mM imidazole. The proteins were then buffer exchanged to 0.1 M sodium bicarbonate buffer, pH 8, for storage, using a PD-10 desalting column (Cytiva #17085101)

### 2.2 Protein Characterization

Protein concentrations were measured using NanoDrop. Sample purity and monomeric size were confirmed using sodium dodecyl sulfate polyacrylamide gel electrophoresis (SDS-PAGE). Samples were mixed (3:1) with 4× Laemmli Sample Buffer (BioRad) and boiled at 95 °C for 2 min before loading onto the BioRad SDS-PAGE gel system. The gel was then stained with Coomassie Blue and imaged using a ChemiDoc XP Gel Imager (BioRad). The purity was quantified using ImageJ. To confirm the identity of the proteins, a western blot was used to confirm the presence of 6xHisTag in the proteins with anti-Penta-His Alexa Fluor 488 Conjugate (Qiagen #35310).

The hydrodynamic size distribution of the proteins was evaluated by dynamic light scattering (DLS) using a Malvern Zetasizer Nano ZS. Samples were diluted to 1 mM and analyzed at a wavelength of 633 nm, with a scattering angle of 173° from a 4 mW He–Ne laser. The settings were specified for 0.1 M NaHCO_3_ as the solvent and protein as the material. Each sample was analyzed in triplicate. For protein stability assessment, the proteins were kept at 4 °C in 0.1 M NaHCO_3_ buffer at pH 8 and then analyzed by DLS after purification and 2 months of storage.

The secondary structure of the proteins was assessed using circular dichroism using a ChiraScan-plus CD spectrometer (Applied Photophysics). Samples were diluted to 1 mM with 0.1 M NaHCO_3_ (pH 8) and added to a 1 mm quartz cuvette. CD signals were collected from 200 to 280 nm and were baselined using a buffer-only signal. We used BeStSel to estimate the secondary structure elements of the constructs from CD data at https://bestsel.elte.hu/index.php (Table S2).^45^

### 2.3 Biolayer Interferometry

SARS-CoV-2 RBD protein was expressed and purified as described by Cheedarla et al. using a Wuhan-Hu-1 strain plasmid (BEI Resources; #NR-52309).^46^ HIS-tagged RBD was diluted to 50 μg/mL in PBS before immobilization onto nickel Octet NTA Biosensors (ForteBio). Protein samples were diluted to 50 μg/mL in PBS and loaded onto a 96-well plate. The association between the samples and RBD was monitored for 1000 s, followed by dissociation in PBS for 1000 s using an Octet RED96e instrument (ForteBio). Tips were regenerated using 10 mM glycine and recharged with 10 mM NiCl_2_ before reloading. A positive control sample was prepared with anti-RBD mAb purchased from GeneTex (#GTX635866). Affinity report was generated in Octet instrument with further data analysis in GraphPad Prism v9 with the following settings: steady state analysis, 1:1 binding model, and global fit.

### 2.4 Neutralization Assay

Pseudovirus production and analysis were done in accordance with the protocol by Nie et al.^47^ Briefly, pseudovirus displaying S protein of three different SARS-CoV-2 variants and containing luciferase gene were prepared as described.^47^ The specific strains used were: Wuhan variant (Wuhan-Hu-1), Delta variant (B.1.617.2), and Omicron variant BA1 (B.1.1.529). For the assay, 0.1 μM protein samples were initially diluted 25-fold with complete DMEM and then subjected to 3-fold serial dilutions. The diluted proteins were mixed with 380 infectious units of pseudovirus/well, and incubated for 1 h at 37 °C. The mixture was introduced to 293T-ACE-2 cells (BEI Resources #NR52511) and incubated for 48 h at 37 °C. Cells were then collected and lysed, after which the luciferase signal was read using a BioTek Synergy H1 plate reader. Relative luminescence unit values were analyzed using GraphPad Prism, in which the half-maximal inhibitory concentration (IC_50_) of each protein was calculated with respect to the no-treatment group through nonlinear regression fits with log scale concentration. Each sample was analyzed in 2 replicates (*n* = 2).

### 2.5 ELISA

ELISA was performed to evaluate the affinity of the modified fusion proteins (HEX-D3-ASA and Bilayer HEX-D3-ASA) to both human serum (hSA) and mouse serum albumin (mSA) at physiological pH. 96-well MaxiSorp plates were coated with 1 μM HEX-D3-ASA or Bilayer HEX-D3-ASA at room temperature (RT) overnight. The plates were washed with Wash Buffer (0.05% Tween 20 v/v in PBS) before each of the following steps. The following day, the plates were blocked with Block Solution (0.05% (v/v) Tween 20 and 1% (w/v) tryptone in PBS) at RT for 1 h. Serial dilutions (2-fold) of mSA or hSA were then added, and the mixtures were incubated at RT for 1 h. Next, diluted primary polyclonal antibody against human and mouse albumin (ThermoFisher PA5-85166) (1:1000 v/v dilution in Block Solution) was introduced and incubated at RT for 1 h. Horseradish peroxidase (HRP)-conjugated goat antirabbit IgG secondary antibody (ThermoFisher 31460) (1:5000 v/v dilution in Block Solution) was then added and incubated in the dark at RT for 1 h. Finally, 50 μL of 3,3′,5,5′-tetramethylbenzidine (TMB) was added to each well to start the reaction with HRP, after which H_2_SO_4_ was added to terminate the enzymatic activity. The absorbance of the wells was measured at 450 and 570 nm to account for the background signal.

After subtracting the background from the 570 nm reading and the negative control, the absorbance data were plotted against albumin concentrations on a log scale using GraphPad Prism 10. The data were then fitted to the One-Site Specific Binding model represented by eq 1, where *Y* is the absorbance at 450 nm, *X* is the albumin concentration, *B*_max_ is the absorbance at the maximum amount of specific binding, and *K*_d_ is the equilibrium dissociation constant. *K*_d_ was reported as a measure of protein avidity to the respective albumins. A two-tailed, unpaired, parametric *t*-test was carried out using GraphPad Prism 10 to evaluate statistical significance.


1 $$Y=\frac{B_{\max}\left[X\right]}{K_{d}+\left[X\right]}$$

### 2.6 *In Vivo* Half-Life and Biodistribution

Animal studies were conducted under protocol A100092, approved by the Georgia Institute of Technology Institutional Animal Care and Use Committee. All animal care and procedures were done according to the University’s Physiological Research Laboratory policies and under ethical guidance following the National Institutes of Health (NIH) guidelines.

Half-life and biodistribution studies were performed in 7-week-old female and male Balb/c mice (Jackson Laboratory) using five proteins: D3 Linear, HEX-D3, HEX-D3-ASA, Bilayer HEX-D3-ASA, and Bilayer HEX-D3. In order to ensure humane treatment of the animals, 2 sets of 4 mice were used for each group to avoid frequent blood sampling from the same animals (*n* = 4). One group of mice was used for 3 and 24 h time points (*n* = 4), the other was used for 1, 48, 72, and 96 h time points (*n* = 4). The mice were kept on an alfalfa-free imaging diet to reduce fluorescence interference during IVIS measurements.

To prepare protein samples for injection, we first removed endotoxins using a Pierce Endotoxin Removal Spin Column (ThermoFisher Scientific #88274) and then labeled them with Alexa Fluor 647 (ThermoFisher Scientific #A20006) before buffer exchanging into endotoxin-free PBS. Proteins were diluted in saline and administered to mice intravenously through the jugular vein at a dose of 6 mg/kg. Blood samples were taken before administration and at 1, 3, 24, 48, 72, and 4 days after administration using the submandibular and saphenous veins into collection tubes with a clot activator (BD #365967). The serum was then separated using centrifugation according to the manufacturer’s directions and diluted 2× before being plated in black 386 well plates. The prepared samples were read using a BioTek Synergy H4 (*E*_x_: 650/9; *E*_m_: 671/9; Gain: 150). Standard curves for each protein were included in each analysis to quantify the sample protein concentration based on linear regression between the fluorescent signal and protein concentration. Half-life calculation was done by fitting the average serum concentration of each set of mice (*n* = 4) against time with one-phase decay nonlinear regression using GraphPad Prism 10 (Figure S7). The average value was used in the fit to account for biological variability and the use of 2 different groups of mice for different time points.

The first set of mice was sacrificed at 24 h, and the other set after 4 days. The liver, spleen, and lungs were then harvested, washed in PBS, and blotted using a low-lint wipe to remove excess blood. They were placed on matte black paper and imaged using the PerkinElmer IVIS system epifluorescence settings for Alexa Fluor 647. Images were analyzed using Living Image Software, and the Auto ROI tool was used to calculate the average radiant efficiency for each organ.

### 2.7 Statistics

Statistical significance between the study groups was calculated using GraphPad Prism 9, with one-way ANOVA with Tukey’s post-hoc multiple comparison analysis. The *p* values are presented as follows: (*) for *p* ≤ 0.05, (**) for *p* ≤ 0.01, (***) for *p* ≤ 0.001, and (****) for *p* ≤ 0.0001. Graphs plotted with error bars are reported as the mean with one standard deviation.

## 3 Results

### 3.1 Design, Expression, and Characterization of DARPin and CC-HEX-Fusion Proteins

Rational protein design principles were used to create fusion proteins of CC-HEX and DARPins. Given that increased valency is likely to sustain binding avidity between the neutralizing molecules and the virus,^35,48,49^ we used CC-HEX to display multiple units of anti-RBD DARPins in close proximity to each other (Figure 1A,B). We tested this design with the three distinct anti-RBD DARPins used in ensovibep,^34^ fused separately onto CC-HEX, resulting in three different therapeutic proteins (HEX-D1, 2, and 3; see sequences in Table S1). For each of these fusion proteins, DARPins were genetically fused to both termini of each monomeric coil of CC-HEX, resulting in a final fusion protein containing 12 DARPins (Figure 1A). Long and flexible glycine-serine linkers (GGGS)_4_ are used as spacers between DARPins and CC-HEX, so as to prevent steric hindrance and increase the number of DARPins that can engage simultaneously.^50^ Another potential advantage of having long linkers is that our fusion proteins could hypothetically bind to multiple viruses at once and facilitate the formation of viral aggregates, which could slow down the spread of the virus^51^ and also improve viral clearance through activation of nonopsonic phagocytic receptors.^52^ As control, we created linear DARPins trimers with glycine-serine linkers (Linear D1–3), mimicking ensovibep’s design (Figure 1A).

**Figure 1:**
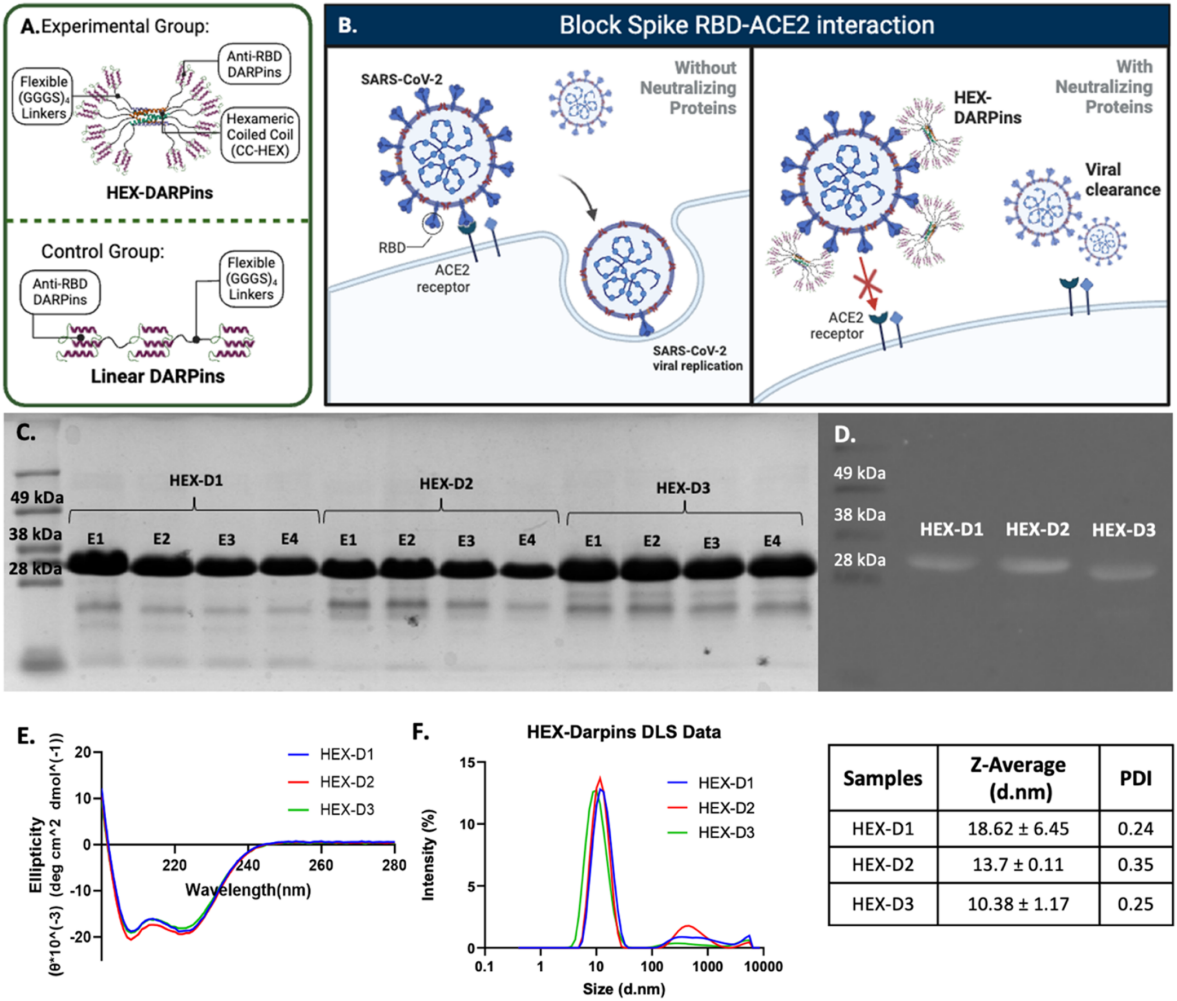
Fusion protein designs and hypothesized mechanism of action: (A) design of HEX-DARPin fusion proteins displaying 12 anti-RBD DARPins with flexible linkers and control Linear DARPin design inspired by ensovibep, with three anti-RBD linked together in a linear fashion. (B) Hypothesized mechanisms of protection against SARS-CoV-2 by HEX-DARPins: HEX-DARPins bind to viral RBD, preventing interaction with the ACE2 receptor and entry to cells. (C) SDS-PAGE of eluted proteins and (D) western blot images confirming purity (88–96%) and successful expression of different HEX-DARPins. (E) Circular dichroism spectra showing a helix-rich secondary structure. (F) DLS spectra and summary data showing the homogeneity and hydrodynamic size of HEX-DARPins in 0.1 M NaHCO_3_.

First, the fusion proteins and controls were recombinantly expressed in *E. coli* and purified using nickel chromatography. A gradient purification method was optimized to effectively eliminate weakly bound species, as we expect 6 repeats of 6X His-Tag in a hexameric configuration to bind very strongly to Ni beads. The purified proteins, with yields ranging from 11 to 22 mg/L for the HEX-DARPins, were then buffer exchanged to 0.1 M NaHCO_3_.

As seen in Figure 1C, the molecular weights of the monomeric units of the fusion proteins matched the theoretical values (28.5–31 kDa), placing the molecular weight of the hexameric constructs at around 250 kDa. We confirmed the identity of the expressed proteins using western blotting, which showed that the proteins in the final eluate contained the recombinant 6X His-Tag (Figure 1D).

The secondary structure of the proteins was characterized by circular dichroism (CD). Spectral minima at 208 and 222 nm were observed, suggesting an α-helix-rich structure (Figure 1E), which was confirmed by BeStSel estimation (Table S2). This is expected considering that both CC-HEX and DARPins are largely made up of α helices, which indicates that the expressed proteins were able to fold correctly. The hydrodynamic size and monodispersity of the proteins were analyzed using dynamic light scattering (DLS). The hexameric fusion proteins have diameters ranging from 10.38 to 18.62 nm, and polydispersity index (PDI) values of 0.24–0.35, indicating the presence of a few monomers or larger aggregates (Figure 1F). In contrast, in the spectra of linear DARPins, smaller monomer and larger aggregate peaks were observed (Figure S2A). The fusion proteins remained soluble in aqueous solution with an unchanged hydrodynamic size for at least 2 months at 4 °C (Figure S2B).

### 3.2 HEX-DARPins Strongly Bind to RBD Regions and Potently Inhibit Viral Entry in a Pseudovirus Model

We evaluated the ability of the proteins to bind to the target, SARS-CoV-2 RBD, using a Biolayer Interferometry (BLI). All molecules bind strongly to soluble SARS-CoV-2 RBD, with *k*_D_ in the subpicomolar range, below the limit of the instrument (Figures 2A, S3, and S4). We then subjected the proteins to a pseudovirus neutralization assay to examine their protective capacity against viral entry. ACE2+ 293T cells were incubated with proteins and pseudoviruses presenting spike proteins of different SARS-CoV-2 variants and a luciferase reporter vector. IC_50_ values are reported in Figure 2B as the concentration of protein at which 50% of the viral load was neutralized. HEX-DARPins proteins are highly potent against all SARS-CoV-2 pseudovirus variants tested (Wuhan, Delta, and Omicron), with IC_50_ values in the low picomolar range. Compared to their linear controls, hexameric DARPin presentation was much more effective at inhibiting viral entry (∼4.1–29.6-fold) for all three DARPins. It should be noted that the IC_50_ values were reported as the molar concentration of each fusion protein, as is standard in the field, and not the amount of anti-RBD DARPin contained in each protein. Nevertheless, even after normalizing for the fact that HEX-DARPins contain 4 times more anti-RBD units than the linear control, they are still better or at least equivalent, depending on the variant of DARPins and pseudovirus.

**Figure 2:**
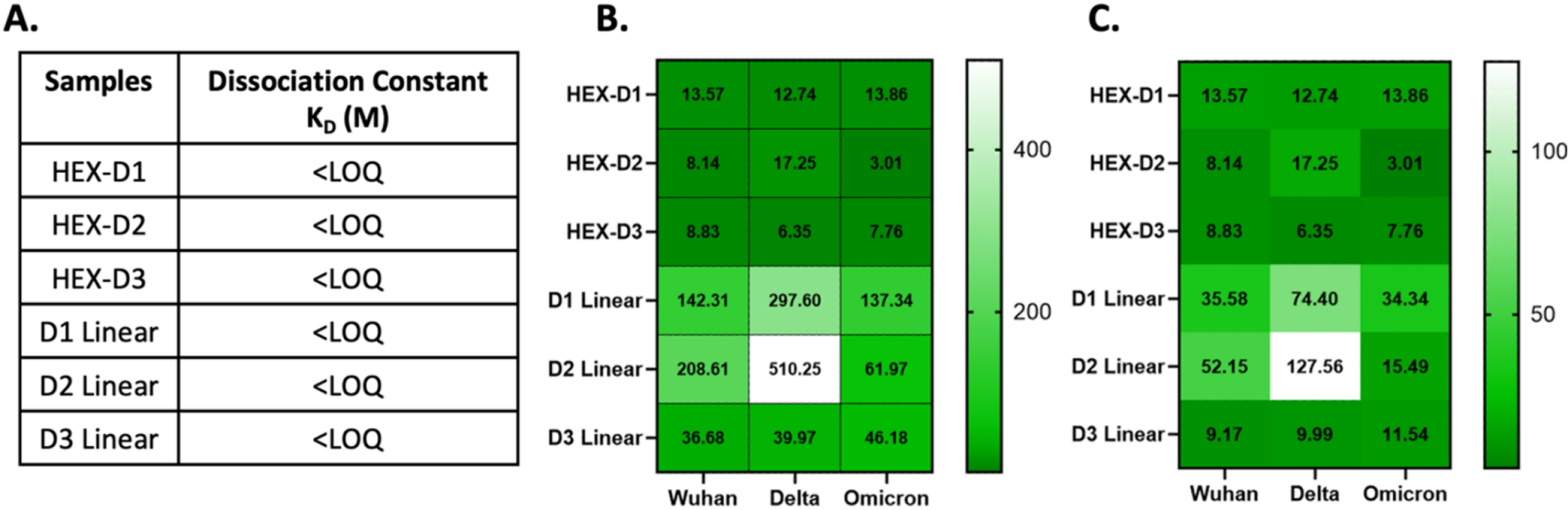
In vitro functional assays of HEX-DARPin fusion proteins. (A) BLI data showing subpicomolar binding avidity for SARS-CoV-2 RBD (LOQ (limit of quantification) = 1 pM). (B) In vitro neutralization potency of HEX-DARPins and controls against different variants of SARS-CoV-2 pseudovirus, reported as IC_50_ concentration of each protein (pM). Each sample was repeated 2 times (*n* = 2). The data fit and standard deviations are shown in Figure S5. (C) Pseudovirus neutralization data after normalized for the number of anti-RBD subunits.

### 3.3 HEX-DARPin Configurations Improve Half-Life and Lung Accumulation in a Mouse Model

The systemic clearance rate is quite a concern for neutralizing therapies, considering that if the drug is cleared before a substantial amount of virus has been neutralized, the remaining virus can continue to rapidly replicate, rendering the therapy ineffective. Although the standard solution to this problem is through the attachment of the synthetic polymer poly­(ethylene glycol) (PEG),^53^ this method comes with issues such as immunogenicity, which counteractively leads to accelerated blood clearance by anti-PEG antibodies.^54^ Albumin hitchhiking, where a protein binds to albumin and thus leverages the recycling mechanisms that keep albumin in the blood, is also a popular method to avoid clearance and has been previously utilized in the design of ensovibep.^34^ We created multiple configurations of HEX-D3 with albumin-binding capacity (Figure 3A), with the goal of extending the protein half-life. HEX-D3 was chosen due to its uniform neutralizing performance across viral variants, both in linear and HEX-fusion forms (Figure 2B). We first switched out the anti-RBD DARPins attached to the N-terminus of CC-HEX for antiserum albumin DARPins, as described previously by Steiner et al.,^44^ to create HEX-D3-ASA (Table S1). However, as there are six albumin-binding molecules adjacent to each other on the fusion protein, there is a concern that they might lead to albumin aggregation, which might result in undesired complications. Further, this design loses 50% of viral-neutralizing domains. Thus, we added an extra layer of anti-RBD DARPins to create steric hindrance to albumin binding and lower the chance of possible aggregation (Bilayer HEX-D3-ASA). We also created a bilayer version of HEX-D3 (Bilayer HEX-D3) to examine the effect of protein size on pharmacokinetics and to serve as a control for Bilayer HEX-D3-ASA. These proteins were successfully expressed in *E. coli* and purified, as shown by SDS-PAGE and western blotting (Figures 3B and S6). The hydrodynamic size and homogeneity of these proteins were confirmed by DLS, with a size of around 20 nm for HEX-D3-ASA and 22–26 nm for Bilayer HEX-D3-ASA and Bilayer HEX-D3 (Figure 3C). As expected, Bilayer HEX-D3-ASA and Bilayer HEX-D3 are slightly larger due to the additional layer of anti-RBD DARPins. It should also be noted that since the constructs are not completely spherical, with a barrel-shaped core and flexible, dynamic “arms”, DLS measurements are likely not precise due to the Stokes–Einstein assumption of sphericity. Although a small population of aggregates is evident in the intensity DLS data, which is weighted toward larger particles, this population is absent in the number plot of the DLS data (Figure S1), indicating that it is a minor component of the protein population. Subsequently, to demonstrate the albumin-binding capacities of these proteins, we used a titration enzyme-linked immunosorbent assay (ELISA) against human and mouse serum albumins (hSA and mSA). Binding to mouse serum albumin was assessed to justify our choice of animal for a later *in vivo* study. The results were fitted to the saturation binding fit function using GraphPad Prism to predict the *K*_d_ between albumin-binding HEX proteins and hSA or mSA. As shown in Figure 3D, all anti-SA HEX proteins have nanomolar affinity for both hSA and mSA, with HEX-D3-ASA binding stronger than that of Bilayer HEX-D3-ASA to both human and mouse targets. This observation agrees with our prediction that an extra “layer” of anti-RBD DARPins will hinder and reduce albumin binding. Additionally, the constructs have higher affinity for hSA, which concurs with previous findings on anti-SA DARPin.^44^ mSA being a less optimal target for anti-SA DARPin could also explain the more pronounced difference in kDa or hindrance effect between the two constructs when tested against mSA.

**Figure 3:**
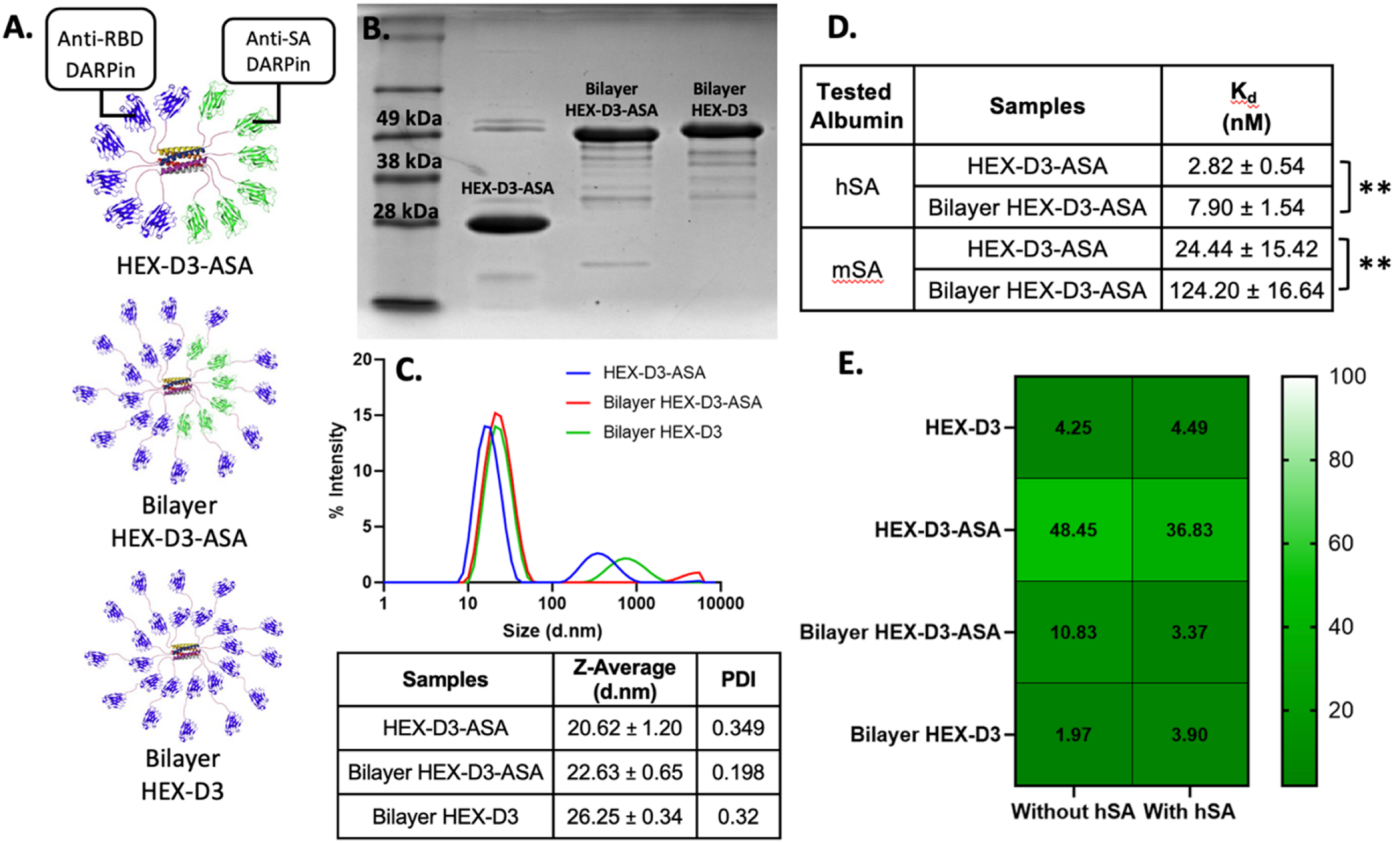
Expression and characterization of albumin-hitchhiking HEX-DARPin variants. (A) Schematic of the different design configurations of HEX-DARPins. (B) SDS-PAGE gel of new HEX-DARPins designs with a purity ranging from 72.4% to 81.6%. (C) DLS spectra and summary data showing the homogeneity and hydrodynamic size of these proteins. (D) Binding kinetics of two albumin-hitchhiking HEX-DARPins to human and mouse serum albumins, calculated using ELISA. Statistical significance was evaluated using a two-tailed, unpaired, parametric *t-*test. The *p* values are presented as follows: (*) for *p* ≤ 0.05, (**) for *p* ≤ 0.01, (***) for *p* ≤ 0.001, and (****) for *p* ≤ 0.0001. (E) Neutralization efficiency of HEX-DARPin designs against Wuhan SARS-CoV-2 pseudovirus in the absence/presence of human serum albumin. IC_50_ values are reported in pM.

We also tested the neutralization efficiencies of HEX-D3-ASA and Bilayer HEX-D3-ASA before and after their binding to albumin. Interestingly, as observed in Figure 3E, both proteins performed better in the presence of hSA, which could be explained by the observed SARS-CoV-2 scavenger function of hSA in the literature.^55^ The potency of HEX-D3-ASA was reduced compared to HEX-D3, as expected due to the lower number of anti-RBD subunits displayed. However, a decrease in potency was not observed with Bilayer HEX-D3-ASA, whose performance was comparable to that of the control in the presence of hSA. This suggests that there is a diminishing return between potency and the number of anti-RBD DARPin units on CC-HEX through tandem linkers, as in the current design of Bilayer HEX-D3. With two layers of anti-RBD DARPins, the outermost layer is likely to create steric hindrance for the inner layer, decreasing its ability to associate with the virus, thus explaining this observation.

The pharmacokinetics and biodistribution of the fusion proteins were examined *in vivo* using Balb/c mice. Proteins were fluorescently labeled and injected intravenously into mice at a 6 mg/kg dose via the jugular vein (*n* = 4). The serum concentrations of each fusion protein were calculated from the fluorescent signals at 1, 3, and 24 h (Figure 4A). As seen in Figure 4A, HEX-D3-ASA consistently showed the highest serum concentration up to 24 h. Indeed, proteins containing anti-albumin DARPins had a much longer half-life compared to their counterparts, ∼13–15 h compared to ∼1 h in mice, respectively (Figure 4B). Additionally, we observed a positive correlation between the size of the molecules and the amount of protein remaining in the bloodstream during the first hour after injection (Figure 4A). However, the effect of albumin binding dominates the effect of size. An extra layer of anti-RBD DARPins increases the size but dampens the protective effect of anti-albumin DARPins against clearance by sterically hindering albumin binding, as shown by comparing HEX-D3-ASA and Bilayer HEX-D3-ASA.

**Figure 4:**
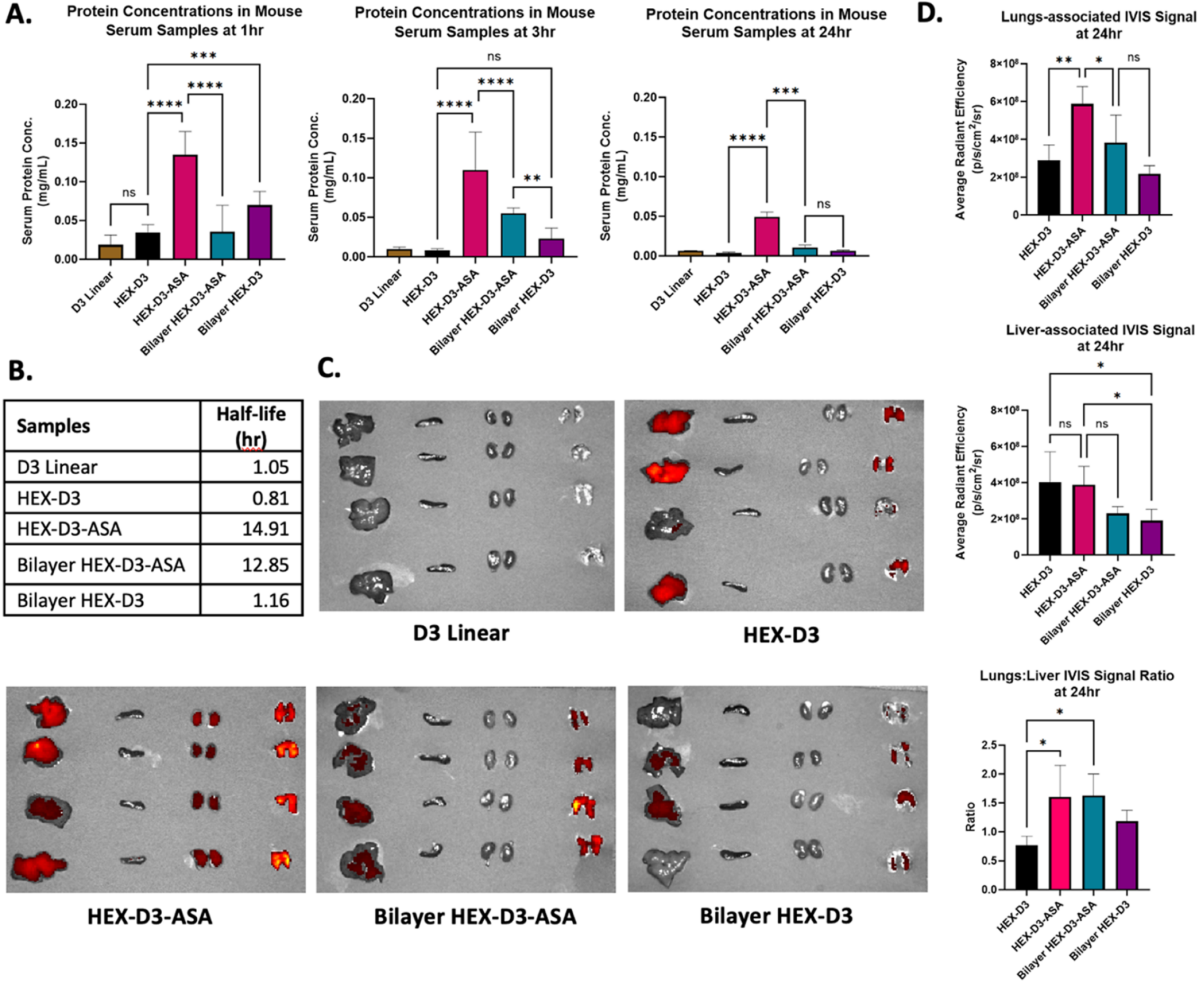
*In vivo* pharmacokinetics and biodistribution evaluation of HEX-DARPin designs in mice. (A) Serum concentrations of different fusion proteins at various time points (*n* = 4). (B) Calculated half-lives of fusion proteins in mice (h). (C) IVIS images of mouse organs after 24 h (left to right: liver, spleen, kidneys, and lungs). (D) Quantification of average fluorescent signals in the lungs and liver, as well as the signal ratio between the two organs (*n* = 4). All error bars show the standard deviation of the data. Statistical significance between the study groups was calculated using one-way ANOVA with Tukey’s post-hoc multiple comparison analysis. The *p* values are presented as follows: (*) for *p* ≤ 0.05, (**) for *p* ≤ 0.01, (***) for *p* ≤ 0.001, and (****) for *p* ≤ 0.0001.

To examine the biodistribution of HEX proteins in mice, fluorescent images of the liver, spleen, and lungs were taken 24 h post-injection. Figure 4C shows that all groups with HEX-fusion proteins have fluorescent signal accumulation in both the lungs and liver, while the Linear D3 group does not have a signal in any organs, likely due to its rapid clearance. Interestingly, we observed that lung accumulation improves greatly with the HEX-D3-ASA design compared to HEX-D3. This observation holds true even when considering the signal ratio between the liver and lungs (Figure 4D), which shows that the accumulation of HEX-D3-ASA in the lungs is ∼1.5 times more than that in the liver. The observed lung accumulation was promising, considering that most nanoparticles administered through systemic injection routes end up largely in the clearance organs, such as the liver and kidneys, and not the lungs.^56^ The lungs are highly vascularized;^57^ thus, as HEX-D3-ASA persists longer in the bloodstream through albumin binding, a higher concentration was sustained in the lungs compared to other groups, even without the inclusion of any lung-targeting moieties. High vascularization of the kidneys could also be responsible for the kidney signal observed in the HEX-D3-ASA group (Figure 4C) though albumin-recycling transcytosis during renal clearance could also be a factor.^58^

## 4 Discussion

Even with great advancements in disease control due to vaccination efforts, COVID-19 remains unpredictable, with great shifts in variants of concern continuing to make people sick.^59^ In severe cases, the viral load overwhelms the immune system and creates a cytokine storm that could lead to respiratory failure and ultimately death.^3^ Early intervention with COVID treatment might dampen the infection’s severity and help avoid long-term effects.^60–62^ To address this need, we created fusion proteins, HEX-DARPins, that can be efficiently produced in bacteria while still providing a highly potent neutralization capability against several variants of SARS-CoV-2. Bacterial expression systems are relatively simple and rapid,^63^ potentially making treatment more accessible and drug development faster, which is crucial in developing therapeutics against mutation-prone, widespread viruses like SARS-CoV-2.

The main therapeutic component of our fusion protein is a small and simple antibody-mimetic protein known as DARPin. Despite comparable affinity and specificity to that of mAbs, small affinity proteins like DARPins often underperform when used by themselves in soluble form.^34,35^ In addition to very rapid clearance, they lack the physical size and valency needed to facilitate effective neutralization. In fact, it is well-known that even for larger molecules like antibodies, single binding interactions between the therapeutic and the virus are often not sufficient for protection.^35^ Additionally, new variants of the virus could diminish the neutralizing potency of even the most broad-protecting mAbs due to mutations in the binding site that decrease the affinity between the neutralizing molecules and the virus.^59,64^ By increasing the multivalency of the therapeutics, both of these concerns could be addressed. Displaying multiple adjacent DARPins improves the probability of multiple binding interactions by increasing the local ligand concentration after the first binding event,^36^ while also increasing the size of the fusion protein. Additionally, multivalency promotes avidity, which allows the conservation of neutralizing potency even when the affinity of the soluble DARPin is weaker toward new variants of the virus.^65^ This informed the rational design of fusion proteins, with a hexameric coiled-coil nanocarrier enabling the flexible display of 12 anti-RBD DARPins in proximity to each other. The modular nature of CC-HEX allows for simple rational design, bacterial production, and self-assembly without downstream fabrication steps.^42,65^ HEX-DARPins demonstrated a potent protective effect in the SARS-CoV-2 pseudovirus model, with IC50 values in the low picomolar range. This performance places HEX-DARPins on par with, if not better than, the most potent mAbs^64^ and ensovibep,^34^ especially considering that only one type of anti-RBD DARPin was displayed on each CC-HEX complex. The improved neutralization potency observed for HEX-DARPins compared to linear controls showed that the multivalent designs better engaged with the virus. We attribute this to the long and flexible glycine-serine linkers (GGGS)_4_ used as spacers between DARPins and CC-HEX to prevent steric hindrance and increase the number of binding domains that can engage simultaneously on a single virus or multiple viruses. Although we did not quantify the number of anti-RBD domains that can bind to the virus simultaneously, previous CC-HEX constructs with identical linkers to 12 Fc-binding domains exhibited full saturation of binding sites with soluble antibodies.^66^ Although it is likely that nonbilayer HEX-DARPins would similarly saturate with soluble RBD due to viral curvature and size, as well as trimeric RBD display on viral surfaces, it is unlikely that all binding domains are engaged during virus neutralization. We also demonstrated broad neutralization with minimal loss of potency against the multiple SARS-CoV-2 variants. In addition to improved avidity, the enhanced potency could be explained by the combined effect of both prolonged inhibition and a hypothetical increase in viral aggregates, as seen in other cases of multimeric neutralizing molecules.^51,67^ We also observed a diminishing benefit on the number of binding units included in tandem in the fusion protein on neutralization efficacy, when comparing the IC50 values of D3 Linear, HEX-D3-ASA, HEX-D3, and Bilayer HEX-D3. This observation suggests that there is a trade-off with the inclusion of more binding units in a linear fashion on each linker “arm”, which could be attributed to spatial distribution and hindrance, limiting the number of RBD sites or viruses that one monomer or one complex can interact with at a time. Another explanation could be the hexavalent limit and circular spatial arrangement of binding units that maximize the valency effect on binding strength in the low-valency regime, as reported previously,^36^ which resembles the DARPin arrangement at either terminus of CC-HEX.

Drug pharmacokinetics is also an important factor during development, as it determines the dosage and frequency of administration, which in turn affects the cost and patient compliance. Albumin derives its long half-life from a recycling mechanism facilitated by its binding to the neonatal Fc receptor (FcRn), which has been leveraged to lengthen the half-life of drugs through a hitchhiking approach.^44,68–70^ We thus designed multiple configurations of HEX-DARPins with albumin-binding ability and studied the effect of different design choices on the protein serum half-life. When injected intravenously into mice, the inclusion of anti-albumin units in both HEX-D3-ASA and Bilayer HEX-D3-ASA allowed the proteins to persist longer in circulation, compared to their respective controls. Other design factors, like increased molecular weight and valency, did not have as strong an effect on the clearance rate as albumin binding, and their effects were short-lived. We also confirmed that much more of the injected fusion protein was distributed to the lungs compared to other organs, which is ideal considering that the lungs are the main infection site of SARS-CoV-2.^3^

## 5 Conclusions

In this work, we created HEX-DARPin fusion proteins with picomolar potency and broad neutralization against SARS-CoV-2 variants. These proteins were efficiently expressed in *E. coli* and were stable at 4 °C for at least 2 months. We also demonstrated significantly increased serum persistence and lung bioavailability of hexameric protein assemblies in a mouse model through albumin hitchhiking. Ultimately, these results show that CC-HEX is an effective platform for multivalent binding protein presentation, where the modular nature can be easily leveraged to create new potent drugs, not just with DARPins but with any or multiple types of binding domains, and controlled modification for hypothesis testing.

In the future, alternative local delivery options should be examined for these neutralizing proteins so that they can be used for COVID-19 prevention in addition to being a treatment option. During the initial stages following exposure, SARS-CoV-2 infects the upper airway and then gradually spreads to the lungs, where serious complications often occur.^3,71^ Thus, rapid deposition of neutralizing proteins in the pulmonary tract, such as by nebulization,^72^ could provide a protective effect by neutralizing the virus before establishment of infection in the lungs. Additionally, given that the nasal cavity has the highest expression of ACE2 receptor compared to the rest of the respiratory tract, intranasal delivery could also be of interest by itself or used in conjunction with nebulization.^73^ In the future, we would like to explore the possibility of applying HEX-DARPins fusion proteins as prophylactics, which are highly desirable in the preparation for future pandemics.^74^

## Supplementary Material



## Notes

Sequence of fusion protein, additional DLS spectra, BLI response plot and fit, pseudovirus neutralization data and fit, additional SDS-PAGE gel and CD spectra, and data and fit for *in vivo* serum concentration over time (PDF)
