# Supplementary material for: Engineering a Coiled-Coil Protein for DARPin Presentation as a Potent SARS-CoV‑2 Therapeutic
Source: Biomacromolecules. 2025 Sep 8;26(10):6504–14. doi: 10.1021/acs.biomac.5c00593 (PMC12522135; doi:10.1021/acs.biomac.5c00593)
Supplement: Supplementary file 1 [file bm5c00593_si_001.pdf]

Supporting Information

Engineering a coiled-coil protein for DARPin presentation as a potent SARS-CoV-2 therapeutic

*Linh D. Mai[a], Narayanaiah Cheedarla[c], Siamalan Krishnathan[b], Ishmamul H. Sadab[a], Mikaela A. Gray[a], Wei Lv[a], Anshul Dhankher[a], John D. Roback[c], Andrew S. Neish[c], Julie A. Champion[a]**

[a] School of Chemical and Biomolecular Engineering, Georgia Institute of Technology, 950 Atlantic Dr NW, Atlanta, GA 30332, United States

[b] School of Materials Science and Engineering, Georgia Institute of Technology, 771 Ferst Drive, Atlanta, GA 30332, United States

[c] School of Medicine, Emory University, 100 Woodruff Circle, Atlanta, GA 30322, United States

Email: Julie.champion@chbe.gatech.edu

Protein	Design	Amino Acid Sequence
D1 Linear	6xHis -DARPin 1-(GGGS)$_4$ linker -DARPin 1-(GGGS)$_4$ linker -DARPin 1	MGHHHHHHGSDLGKKLLQAARAGQLDEVRELLKAGADVNAKDREGITPLHLAAQHGHLEIVEVLLKAGADVNAKDVWGRTPLHLAAWQGHLEIVEVLLKAGADVNAKDLAGATPLHVAALYGHLEIVEVLLKAGADVNAQDKSGKTPADLAARAGHQDIAEVLQKAAGGGSGGGSGGGSGGGSDLGKKLLQAARAGQLDEVRELLKAGADVNAKDREGITPLHLAAQHGHLEIVEVLLKAGADVNAKDVWGRTPLHLAAWQGHLEIVEVLLKAGADVNAKDLAGATPLHVAALYGHLEIVEVLLKAGADVNAQDKSGKTPADLAARAGHQDIAEVLQKAAGGGSGGGSGGGSGGGSDLGKKLLQAARAGQLDEVRELLKAGADVNAKDREGITPLHLAAQHGHLEIVEVLLKAGADVNAKDVWGRTPLHLAAWQGHLEIVEVLLKAGADVNAKDLAGATPLHVAALYGHLEIVEVLLKAGADVNAQDKSGKTPADLAARAGHQDIAEVLQKAA
D2 Linear	6xHis-DARPin 2-(GGGS)$_4$ linker-DARPin 2-(GGGS)$_4$ linker-DARPin 2)	MGHHHHHHGSDLGKKLLQAARAGQLDEVRELLKAGADVNAKDREGKTPLHVAAQEGHLEIVEVLLKAGADVNAKDVWGRTPLHLAAWIGHLEIVEVLLKAGADVNAKDVSGATPLHAAALHGHLEIVEVLLNAGADVNAQDKSGKTPADLAARAGHQDIAEVLQKAAGGGSGGGSGGGSGGGSDLGKKLLQAARAGQLDEVRELLKAGADVNAKDREGKTPLHVAAQEGHLEIVEVLLKAGADVNAKDVWGRTPLHLAAWIGHLEIVEVLLKAGADVNAKDVSGATPLHAAALHGHLEIVEVLLNAGADVNAQDKSGKTPADLAARAGHQDIAEVLQKAAGGGSGGGSGGGSGGGSDLGKKLLQAARAGQLDEVRELLKAGADVNAKDREGKTPLHVAAQEGHLEIVEVLLKAGADVNAKDVWGRTPLHLAAWIGHLEIVEVLLKAGADVNAKDVSGATPLHAAALHGHLEIVEVLLNAGADVNAQDKSGKTPADLAARAGHQDIAEVLQKAA

D3 Linear	6xHis-DARPin 3-(GGGS)$_4$ linker -DARPin 3-(GGGS)$_4$ linker -DARPin 3	MGHHHHHHGSDLGKKLLQAARAGQLDEVRELLKAGADVNAKDQEGITPLHVAAHQGHLEIVEVLLKAGADVNAKDVWGRTPLHLAAWRGHLEIVEVLLKAGADVNAKDHAGATPLHAAALSGHLEIVEVLLKAGADVNAQDKSGKTPADLAARAGHQDIAEVLQKAAGGGSGGGSGGGSGGGSDLGKKLLQAARAGQLDEVRELLKAGADVNAKDQEGITPLHVAAHQGHLEIVEVLLKAGADVNAKDVWGRTPLHLAAWRGHLEIVEVLLKAGADVNAKDHAGATPLHAAALSGHLEIVEVLLKAGADVNAQDKSGKTPADLAARAGHQDIAEVLQKAAGGGSGGGSGGGSGGGSDLGKKLLQAARAGQLDEVRELLKAGADVNAKDQEGITPLHVAAHQGHLEIVEVLLKAGADVNAKDVWGRTPLHLAAWRGHLEIVEVLLKAGADVNAKDHAGATPLHAAALSGHLEIVEVLLKAGADVNAQDKSGKTPADLAARAGHQDIAEVLQKAA
HEX-D1	6xHis tag-DARPin 1-(GGGS)$_4$ linker -HEX-(GGGS)$_4$ linker-DARPin 1	MGHHHHHHGSDLGKKLLQAARAGQLDEVRELLKAGADVNAKDREGITPLHLAAQHGHLEIVEVLLKAGADVNAKDVWGRTPLHLAAWQGHLEIVEVLLKAGADVNAKDLAGATPLHVAALYGHLEIVEVLLKAGADVNAQDKSGKTPADLAARAGHQDIAEVLQKAAGGGSGGGSGGGSGGGSGELKAIAQELKAIAKELKAIAWELKAIAQGAGGGGSGGGSGGGSGGGSDLGKKLLQAARAGQLDEVRELLKAGADVNAKDREGITPLHLAAQHGHLEIVEVLLKAGADVNAKDVWGRTPLHLAAWQGHLEIVEVLLKAGADVNAKDLAGATPLHVAALYGHLEIVEVLLKAGADVNAQDKSGKTPADLAARAGHQDIAEVLQKAA
HEX-D2	6xHis-DARPin 2-(GGGS)$_4$ linker -HEX-(GGGS)$_4$ linker -DARPin 2	MGHHHHHHGSDLGKKLLQAARAGQLDEVRELLKAGADVNAKDREGKTPLHVAAQEGHLEIVEVLLKAGADVNAKDVWGRTPLHLAAWIGHLEIVEVLLKAGADVNAKDVSGATPLHAAALHGHLEIVEVLLNAGADVNAQDKSGKTPADLAARAGHQDIAEVLQKAAGGGSGGGSGGGSGGGSGELKAIAQELKAIAKELKAIAWELKAIAQGAGGGGSGGGSGGGSGGGSDLGKKLLQAARAGQLDEVRELLKAGADVNAKDREGKTPLHVAAQEGHLEIVEVLLKAGADVNAKDVWGRTPLHLAAWIGHLEIVEVLLKAGADVNAKDVSGATPLHAAALHGHLEIVEVLLNAGADVNAQDKSGKTPADLAARAGHQDIAEVLQKAA
HEX-D3	6xHis-DARPin 3-(GGGS)$_4$ linker -HEX-(GGGS)$_4$ linker -DARPin 3	MGHHHHHHGSDLGKKLLQAARAGQLDEVRELLKAGADVNAKDQEGITPLHVAAHQGHLEIVEVLLKAGADVNAKDVWGRTPLHLAAWRGHLEIVEVLLKAGADVNAKDHAGATPLHAAALSGHLEIVEVLLKAGADVNAQDKSGKTPADLAARAGHQDIAEVLQKAAGGGSGGGSGGGSGGGSGELKAIAQELKAIAKELKAIAWELKAIAQGAGGGGSGGGSGGGSGGGSDLGKKLLQAARAGQLDEVRELLKAGADVNAKDQEGITPLHVAAHQGHLEIVEVLLKAGADVNAKDVWGRTPLHLAAWRGHLEIVEVLLKAGADVNAKDHAGATPLHAAALSGHLEIVEVLLKAGADVNAQDKSGKTPADLAARAGHQDIAEVLQKAA
HEX-D3-ASA	6xHis-antiSA-(GGGS)$_4$ linker -HEX- (GGGS)$_4$ linker - DARPin 3	MGHHHHHHGSDLGKKLLEAARAGQDDEVRELLKAGADVNAKDYFSHTPLHLAARNGHLKIVEVLLKAGADVNAKDFAGKTPLHLAANEGHLEIVEVLLKAGADVNAQDIFGKTPADIAADAGHEDIAEVLQKAAGGGSGGGSGGGSGGGSGELKAIAQELKAIAKELKAIAWELKAIAQGAGGGGSGGGSGGGSGGGSDL

		GKKLLQAARAGQLDEVRELLKAGADVNAKDQEGITPLHVA AHQGHLEIVEVLLKAGADVNAKDVWGRTPLHLAAWRGHLE IVEVLLKAGADVNAKDHAGATPLHAAALSGHLEIVEVLLKAG ADVNAQDKSGKTPADLAARAGHQDIAEVLQKAA
Bilayer HEX-D3-ASA	6xHis- DARPin 3-$(GGGS)_4$ linker -antiSA-$(GGGS)_4$ linker- HEX- $(GGGS)_4$ linker - DARPin 3-$(GGGS)_4$ linker - DARPin 3	MGHHHHHHGSDLGKKLLQAARAGQLDEVRELLKAGADVN AKDQEGITPLHVAAHQGHLEIVEVLLKAGADVNAKDVWGR TPLHLAAWRGHLEIVEVLLKAGADVNAKDHAGATPLHAAAL SGHLEIVEVLLKAGADVNAQDKSGKTPADLAARAGHQDIAE VLQKAAGGGSGGGSGGGSGGGSDLGKKLLEAARAGQDD EVRELLKAGADVNAKDYFSHTPLHLAARNGHLKIVEVLLKA GADVNAKDFAGKTPLHLAANEGHLEIVEVLLKAGADVNAQ DIFGKTPADIAADAGHEDIAEVLQKAAGGGSGGGSGGGSG GGSGELKAIAQELKAIAKELKAIAWELKAIAQGAGGGGSGG GSGGGSGGGSDLGKKLLQAARAGQLDEVRELLKAGADVN AKDQEGITPLHVAAHQGHLEIVEVLLKAGADVNAKDVWGR TPLHLAAWRGHLEIVEVLLKAGADVNAKDHAGATPLHAAAL SGHLEIVEVLLKAGADVNAQDKSGKTPADLAARAGHQDIAE VLQKAAGGGSGGGSGGGSGGGSDLGKKLLQAARAGQLD EVRELLKAGADVNAKDQEGITPLHVAAHQGHLEIVEVLLKA GADVNAKDVWGRTPLHLAAWRGHLEIVEVLLKAGADVNAK DHAGATPLHAAALSGHLEIVEVLLKAGADVNAQDKSGKTP ADLAARAGHQDIAEVLQKAA
Bilayer HEX-D3	6xHis- DARPin 3-$(GGGS)_4$ linker – DARPin 3 - $(GGGS)_4$ linker -HEX-$(GGGS)_4$ linker - DARPin 3-$(GGGS)_4$ linker - DARPin 3	MGHHHHHHGSDLGKKLLQAARAGQLDEVRELLKAGADVN AKDQEGITPLHVAAHQGHLEIVEVLLKAGADVNAKDVWGR TPLHLAAWRGHLEIVEVLLKAGADVNAKDHAGATPLHAAAL SGHLEIVEVLLKAGADVNAQDKSGKTPADLAARAGHQDIAE VLQKAAGGGSGGGSGGGSGGGSDLGKKLLQAARAGQLD EVRELLKAGADVNAKDQEGITPLHVAAHQGHLEIVEVLLKA GADVNAKDVWGRTPLHLAAWRGHLEIVEVLLKAGADVNAK DHAGATPLHAAALSGHLEIVEVLLKAGADVNAQDKSGKTP ADLAARAGHQDIAEVLQKAAGGGSGGGSGGGSGGGSGE LKAIAQELKAIAKELKAIAWELKAIAQGAGGGGSGGGSGGG SGGGSDLGKKLLQAARAGQLDEVRELLKAGADVNAKDQE GITPLHVAAHQGHLEIVEVLLKAGADVNAKDVWGRTPLHLA AWRGHLEIVEVLLKAGADVNAKDHAGATPLHAAALSGHLEI VEVLLKAGADVNAQDKSGKTPADLAARAGHQDIAEVLQKA AGGGSGGGSGGGSGGGSDLGKKLLQAARAGQLDEVREL LKAGADVNAKDQEGITPLHVAAHQGHLEIVEVLLKAGADVN AKDVWGRTPLHLAAWRGHLEIVEVLLKAGADVNAKDHAGA TPLHAAALSGHLEIVEVLLKAGADVNAQDKSGKTPADLAAR AGHQDIAEVLQKAA

Table S1: Design and amino acid sequences of all fusion proteins.

	HEX-D1	HEX-D2	HEX-D3
Helix (%)	96.2	97.3	98.7
Beta Turn (%)	2.3	2.7	1.3

Table S2: Estimation of secondary structure for HEX-DARPins constructs using BeStSel.

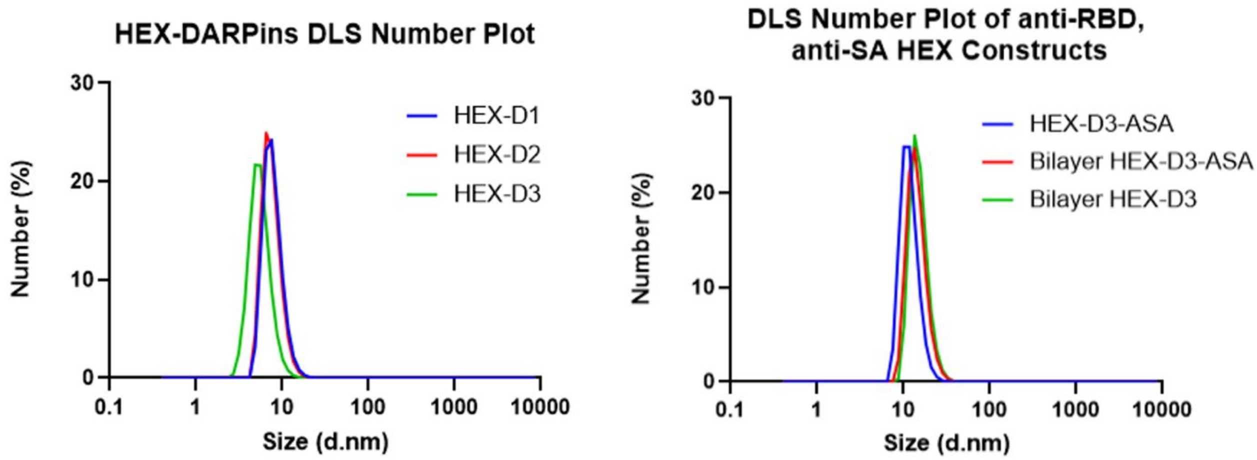

Figure S1: DLS number plots of protein constructs.

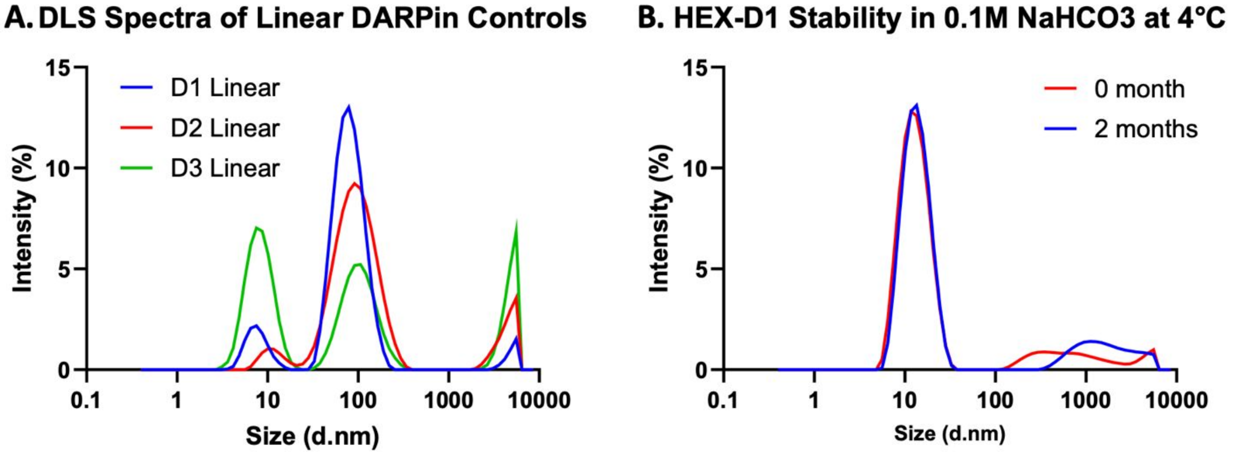

Figure S2: (A) DLS spectra for linear DARPin controls; (B) Stability data of HEX-D1 when stored in 0.1 M $NaHCO_3$ at 4˚C

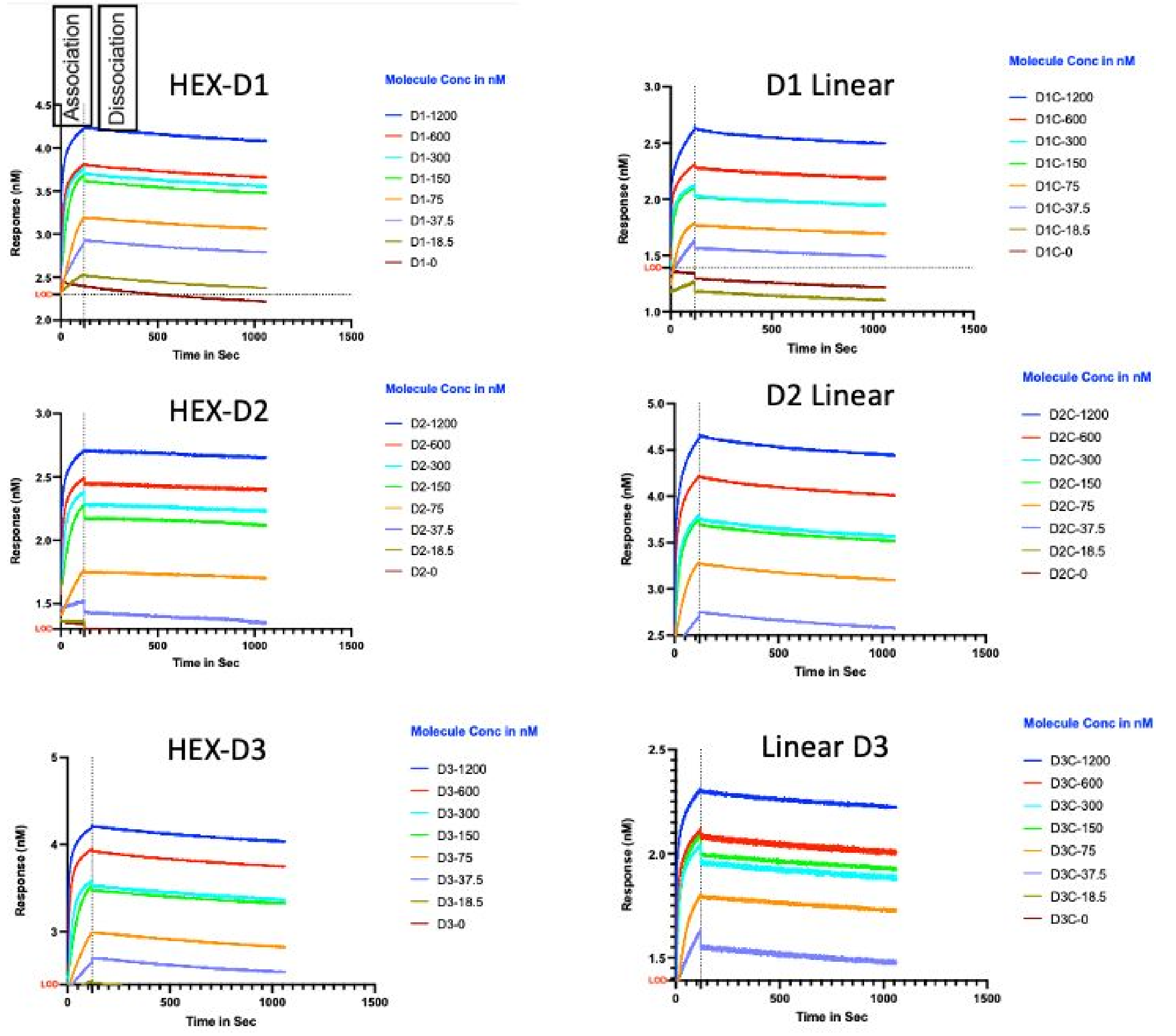

Figure S3: BLI response plots used to calculate binding affinities (listed in Fig. 2A) against SARS-CoV-2 RBD.

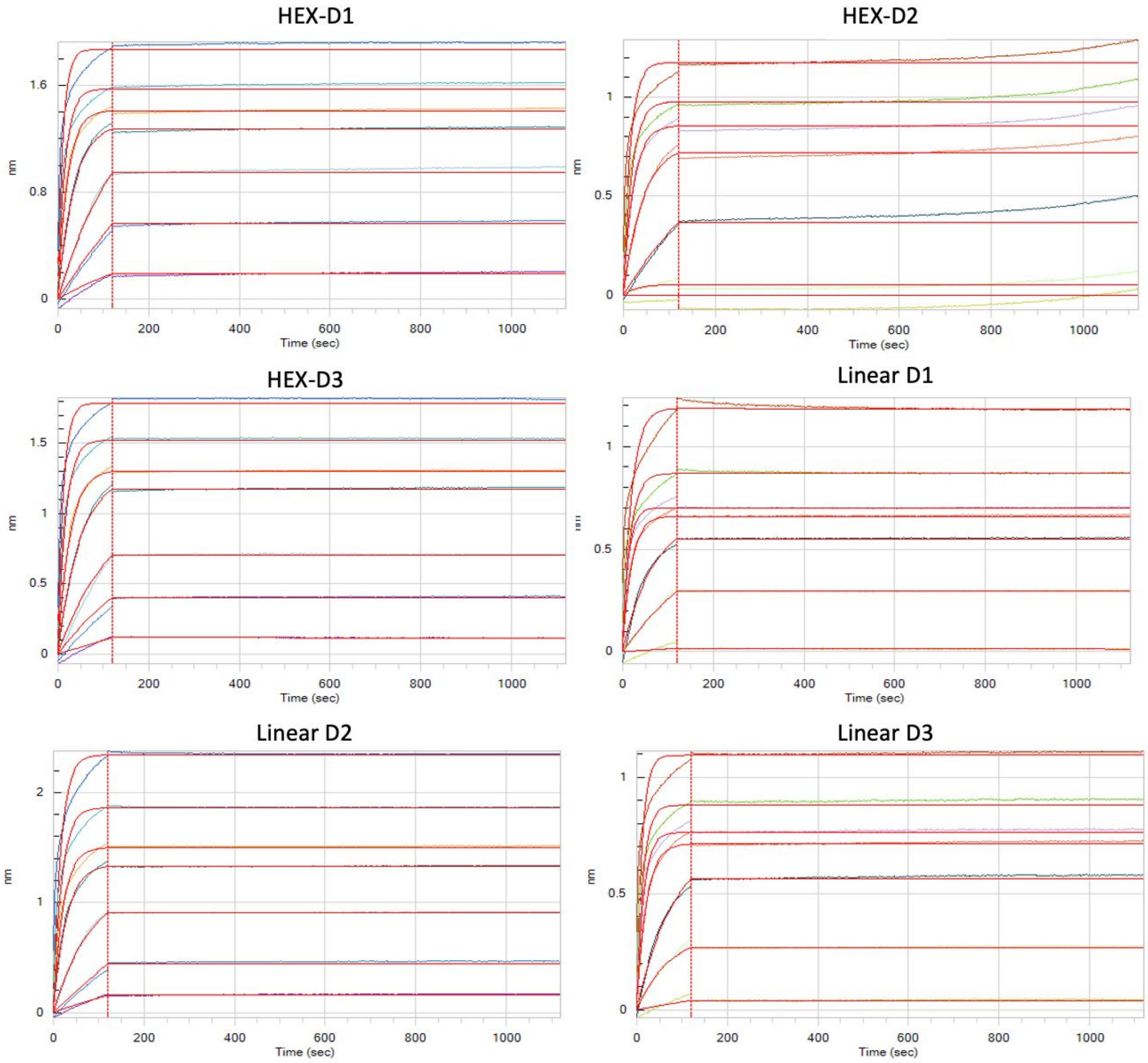

Chi-squared Values of Fit						
**Concentration (nM)**	**HEX-D1**	**HEX-D2**	**HEX-D3**	**Linear D1**	**Linear D2**	**Linear D3**
1200	4.57	3.18	3.04	2.34	5.63	1.61
600	2.23	1.82	0.61	0.78	2.15	0.89
300	0.24	1.21	0.08	0.15	0.71	0.21
150	0.21	0.96	0.14	0.04	0.13	0.13
75	0.71	3.11	0.33	0.07	0.06	0.16
37.5	0.35	0.58	0.55	0.02	0.47	0.05
18.5	0.32	3.25	0.14	0.09	0.13	0.05

Figure S4: (A) Plots of BLI data after baseline subtraction and alignment and fitting for each protein construct against SARS-CoV-2 RBD (Wuhan strain). (B) Chi-squared values of fit.

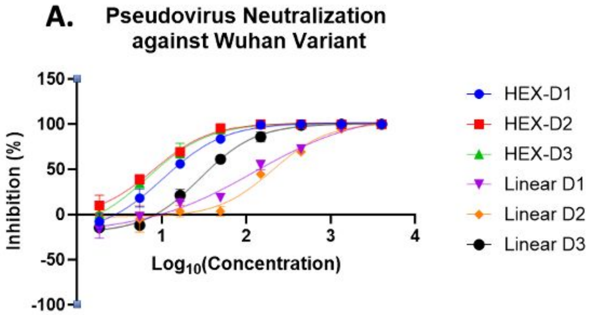

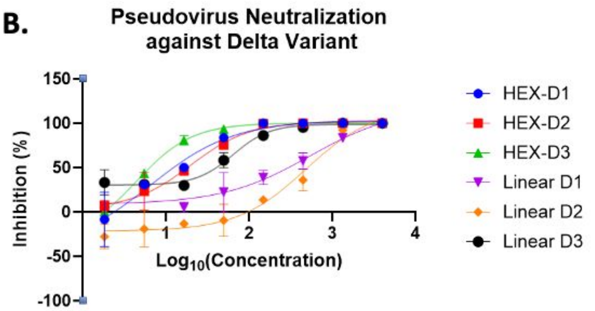

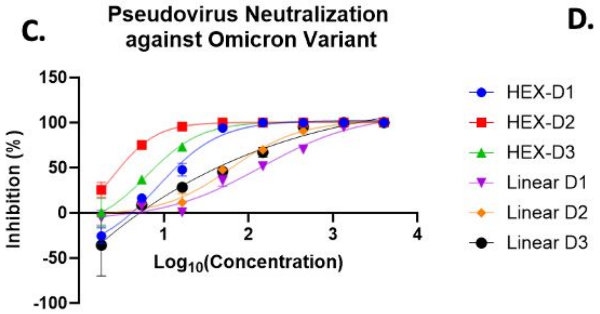

D.

Samples	Wuhan	Delta	Omicron
HEX-D1	13.57 ± 2.37	12.74 ± 0.66	13.86 ± 0.97
HEX-D2	8.14 ± 0.16	17.25 ± 4.26	3.01 ± 0.17
HEX-D3	8.83 ± 2.03	6.36 ± 0.30	7.76 ± 0.46
D1 Linear	142.31 ± 26.89	297.60 ± 144.21	137.34 ± 26.49
D2 Linear	208.61 ± 10.13	510.25 ± 86.69	61.97 ± 5.56
D3 Linear	9.17 ± 1.31	39.97 ± 12.27	46.18 ± 17.64

Figure S5: Pseudovirus neutralization of HEX-DARPins and controls against (A) Wuhan strain; (B) Delta strain; (C) Omicron strain and (D) Fitted IC50 values and standard deviation in pM. Each condition was repeated 2 times.

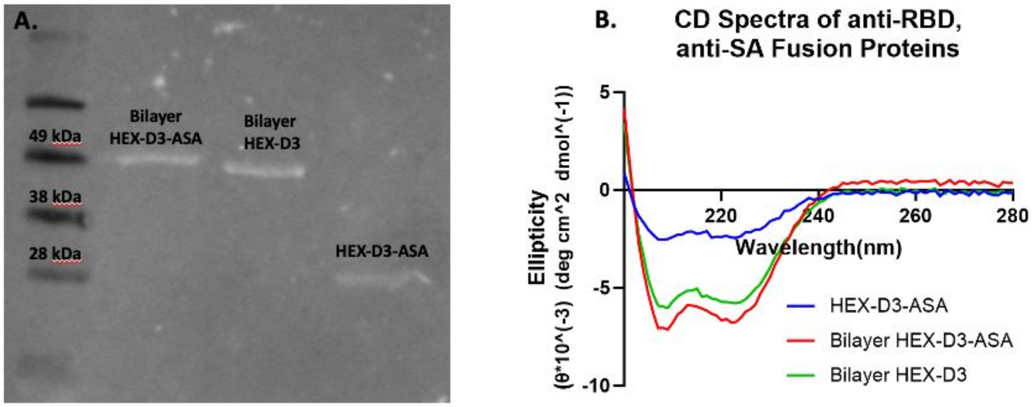

Figure S6: Characterization data for albumin binding and bilayer designs of HEX-DARPins. (A) Western blot detecting 6xHis-tag. (B) CD spectra.

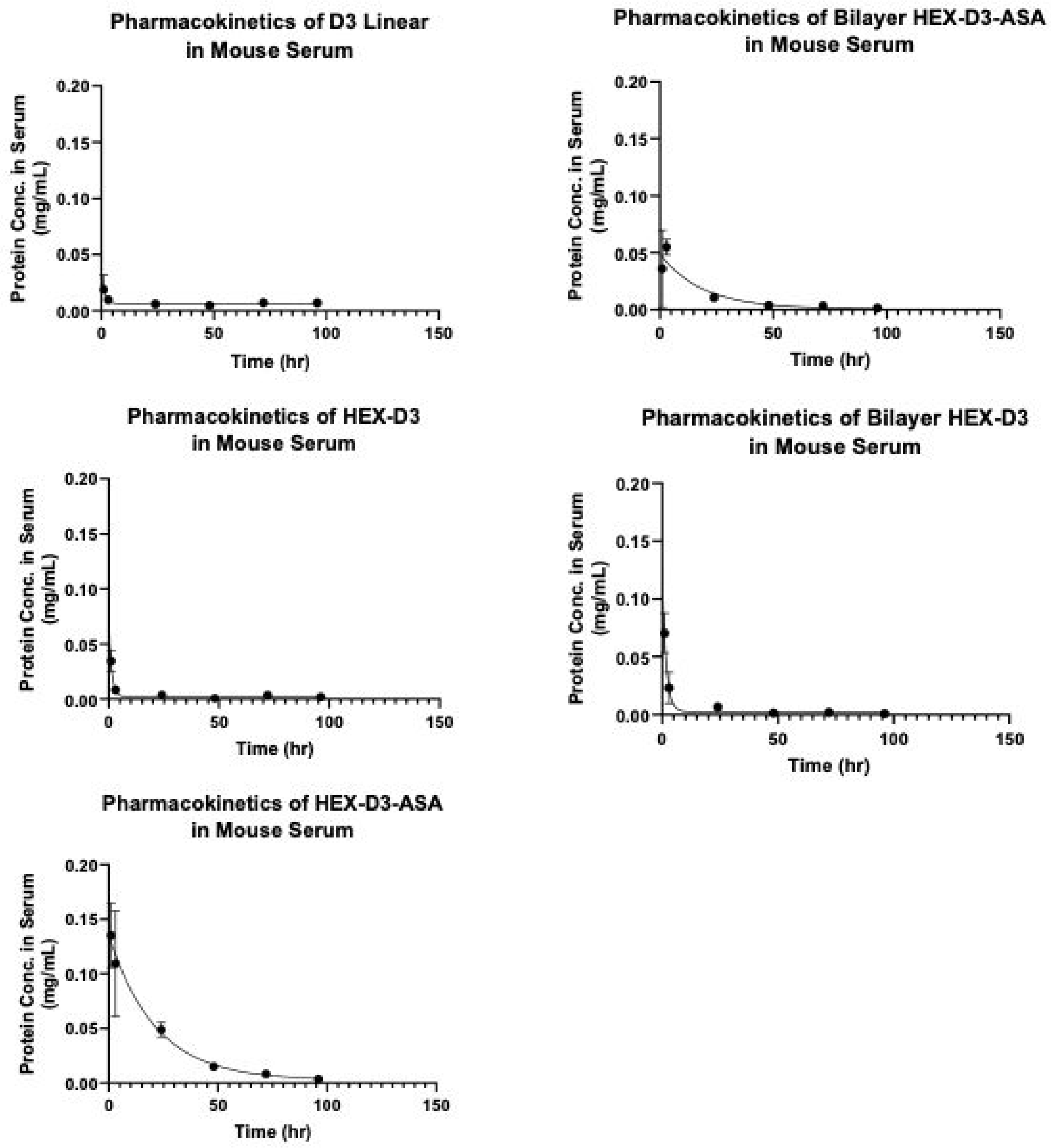

Figure S7: Serum concentration of fusion proteins over time *in vivo*.
